# Isocyanides as a Chemical Tool for Identifying [Fe]‐Hydrogenase‐Dependent Methanogenesis

**DOI:** 10.1002/cbic.70535

**Published:** 2026-09-14

**Authors:** Shunsuke Nomura, Seigo Shima

**Affiliations:** ^1^ Microbial Protein Structure Group, Max Planck Institute for Terrestrial Microbiology Marburg Germany

**Keywords:** bioinorganic chemistry, enzyme catalysis, inhibitors, isocyanide ligands, metalloenzymes

## Abstract

In the hydrogenotrophic methanogens cultivated under nickel‐sufficient standard medium (~5 µM Ni^2+^), two cytosolic [NiFe]‐hydrogenases, F_420_‐reducing hydrogenase (Frh) and heterodisulfide reductase (Hdr)‐associating hydrogenase (Mvh), mainly provide electrons to the methanogenic pathway. In *Methanothermobacter marburgensis* under strictly nickel‐limited conditions (< 50 nM Ni^2+^), which are encountered in certain natural environments, Frh and Mvh are strongly downregulated. Under these conditions, a coupled reaction with [Fe]‐hydrogenase (Hmd) and F_420_‐dependent methylene‐tetrahydromethanopterin dehydrogenase (Mtd) substitutes for the function of Frh, where F_420_‐dependent electron‐donating protein (Elp) complexes with Hdr and donates electrons from the reduced form of F_420_ to Hdr. Thus, Hmd mainly provides electrons to methanogenesis in nickel‐limited environments. In this study, to evaluate the potential of isocyanides as an Hmd‐specific chemical tool to probe this pathway, we determined their inhibitory effects on the H_2_‐dependent F_420_‐reducing activity in the cell extract and on the in vitro methanogenesis reaction in a cell extract and in a cell suspension. Isocyanides specifically inhibited the activities of the samples from nickel‐limited cells. The inhibitory effect on the cell suspension was strengthened by the use of a hydrophobic isocyanide. These results demonstrate their potential as a chemical tool for identifying in situ Hmd‐dependent methanogenesis activity in natural environmental samples.

## Graphical abstract

In model methanogens, [Fe]‐hydrogenase (Hmd) substitutes nickel‐dependent hydrogenases under nickel‐limited conditions. We determined the inhibitory effects of the Hmd inhibitor, isocyanides, on methanogenesis in a cell extract and in a cell suspension. These results demonstrate the potential of isocyanides as a chemical tool for identifying Hmd‐dependent methanogenesis in natural environmental samples.

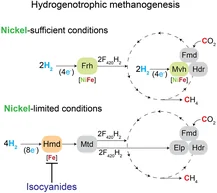

## 1 Introduction

In nature, methanogenic archaea produce large amounts of methane, a greenhouse gas, through their energy metabolism [1]. Most methanogens hydrogenotrophically produce methane by reducing CO_2_ with H_2_ [2]. In hydrogenotrophic methanogens without cytochromes, there are three different types of [NiFe] hydrogenases: heterodisulfide reductase‐associating hydrogenase (Mvh), F_420_‐reducing hydrogenase (Frh), and membrane‐bound energy‐converting hydrogenases (Eha and Ehb). In addition, many methanogens without cytochromes possess a nickel‐independent hydrogenase, H_2_‐forming methylene‐tetrahydromethanopterin (methylene‐H_4_MPT) dehydrogenase ([Fe]‐hydrogenase or Hmd) [3].

**SCHEME 1:**
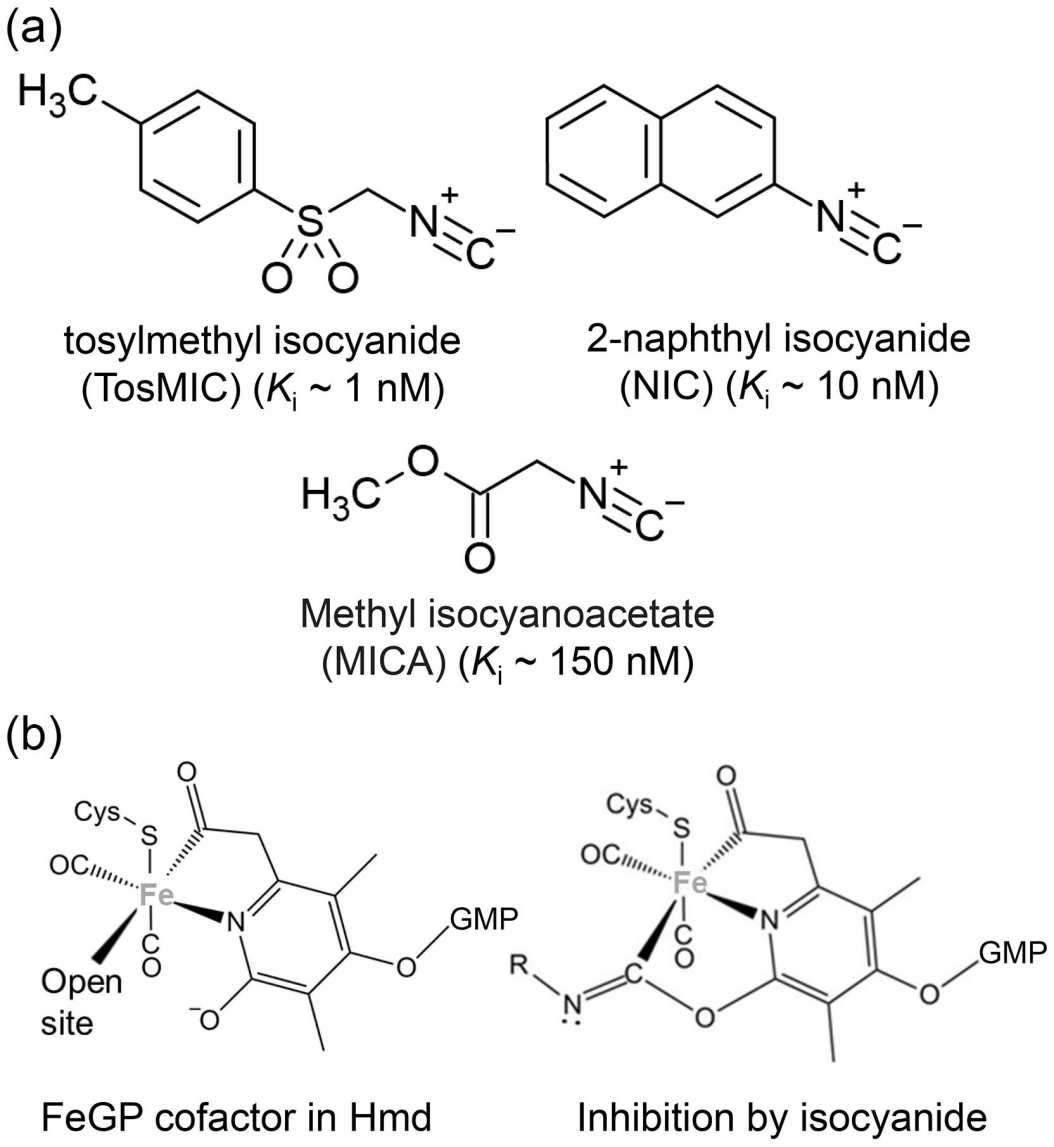
Structure of isocyanides and the FeGP cofactor. (a) Structures of isocyanides with inhibition constants (*K* *_i_*) [7, 8]. (b) The FeGP cofactor in [Fe]‐hydrogenase and its inhibited form with isocyanide. The open site of the FeGP cofactor is proposed to be bound to H_2_ in the catalytic cycle [9]. Isocyanide carbon is bound to iron and the deprotonated hydroxyl group of the FeGP cofactor in the crystal structure [8]. Panel b was modified from [8] with permission from the publisher. TosMIC forms hydrogen bonds with a threonine residue in Hmd, which may explain why its inhibition constant is lower than that of other isocyanides (Figure S1).

The function of Hmd is to transfer a hydride from H_2_ to methenyl‐tetrahydromethanopterin (methenyl‐H_4_MPT^+^), forming methylene‐H_4_MPT [4, 5]. H_2_ is activated at the iron‐guanylylpyridinol (FeGP) cofactor in this enzyme [6]. Hmd is inhibited by isocyanides with very high affinity (*K*_i_ ~ 1–150 nM), whereas the other types of hydrogenase are not inhibited by isocyanides [7]. In the crystal structure of the inhibited form of Hmd with tosylmethyl isocyanide (TosMIC) or 2‐naphthyl isocyanide (NIC) (Scheme 1), the isocyanide group is bound to the open coordination site of iron of the FeGP cofactor and the hydroxy oxygen of pyridinol (Figure S1) [8].

**FIGURE 1:**
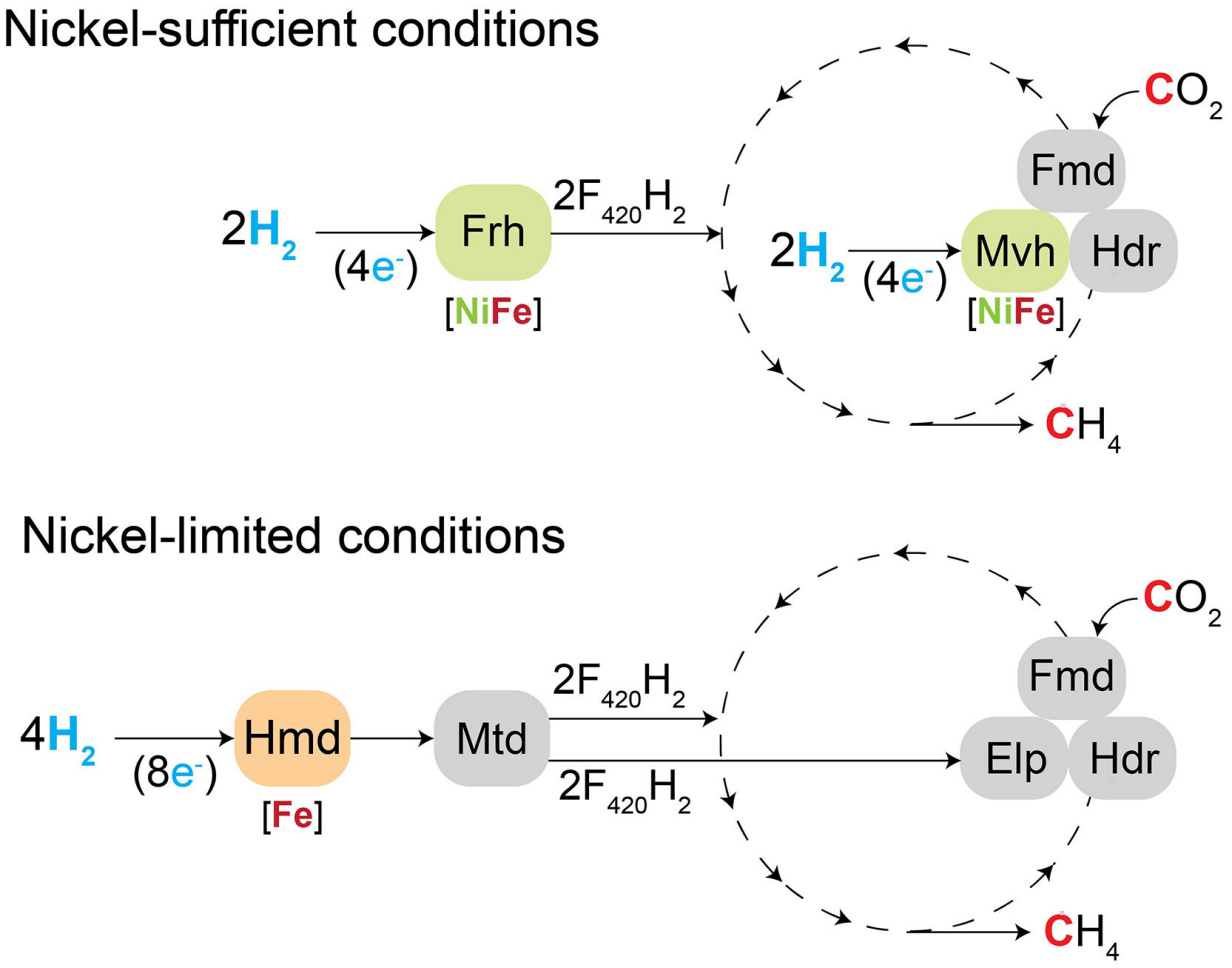
The electron flows in the methanogenic pathway. The circles made of the arrows indicate the methanogenic Wolfe cycle [10], which is closed by the reactions catalyzed by the enzyme complexes involving Hdr. For abbreviations of the enzymes, see text. Under nickel‐sufficient conditions, mainly [NiFe]‐hydrogenases provide eight electrons to the methanogenic pathway: four electrons from Frh via F_420_H_2_ and four electrons from Mvh that form a complex with Hdr and formylmethanofuran dehydrogenase (Fmd). Under nickel‐limited conditions, Hmd mainly provides eight electrons via F_420_H_2_ to the methanogenic pathway, in which Elp forms a complex with Hdr and Fmd [11].

In Figure 1, the electron flows in the methanogenic pathway under nickel‐sufficient and nickel‐limited conditions are shown. Under the standard laboratory culture conditions of a model methanogen, *Methanothermobacter marburgensis*, the medium contains a high concentration of nickel ions (5 µM), which stimulate growth of methanogens because several key enzymes in the methanogenic pathway contain nickel within the prosthetic group. In the standard nickel‐sufficient culture, the cytosolic [NiFe]‐hydrogenases (Frh and Mvh) catalyze the electron‐donating function from H_2_ in the methanogenic pathway [3, 12]. In contrast, nickel‐ion concentrations in natural environments are mainly in the nanomolar range [13, 14, 15, 16]. Nomura et al. reported that under nickel‐limited conditions (< 50 nM Ni^2+^), the production of Frh and Mvh ([NiFe]‐hydrogenases) is strictly downregulated [11, 17], and electron flow in the hydrogenotrophic methanogenesis is dramatically changed. In the altered metabolism, a coupled reaction of Hmd with F_420_‐dependent methylene‐H_4_MPT dehydrogenase (Mtd) (Hmd+Mtd coupled reaction) mediates the reduction of F_420_, which replaces Frh [11]. Mvh is substituted by F_420_‐dependent electron‐donating protein (Elp) [11], which supplies electrons from the reduced form of F_420_ to the electron‐bifurcating reaction, thereby providing low‐potential electrons for the reduction of CO_2_ via diffusible ferredoxin [18] or directly through polyferredoxin to the enzyme catalyzing CO_2_ reduction [11, 19, 20, 21, 22, 23]. Similar changes in electron flow have also been reported in *Methanothermococcus thermolithotrophicus* [11]. Based on the conservation of Hmd and Elp in Class 1 methanogens, it has been proposed that this change is applicable to these methanogens in many nickel‐limited natural environments [13, 14, 15, 16], for example, soils and marine sediments, where Class‐1 methanogens contribute to methanogenesis. However, it was pointed out in the paper that studies of environmental samples with known biologically available nickel concentrations are required to understand the contribution of the non‐nickel Hmd system in nature [11]. For this aim, techniques such as metaproteomics and metatranscriptomics could be applied; however, simpler detection methods are required for the analysis of environmental samples collected from a wide area.

Here, we demonstrate that isocyanides act as effective inhibitors of methanogenesis in both cell extracts and cell suspensions, offering a potential selective tool for identifying Hmd‐dependent methanogenesis in natural environmental samples. It is expected that this approach will be utilized in research aimed at elucidating the contribution of non‐nickel and Hmd‐dependent systems in nature based on the degree of inhibition of methane production reactions in environmental samples with known nickel‐bioavailability.

## 2 Results and Discussion

### 2.1 Characterization of H_2_‐Dependent F_420_ Reducing Activity Catalyzed by Hmd in Cell Extracts

**FIGURE 2:**
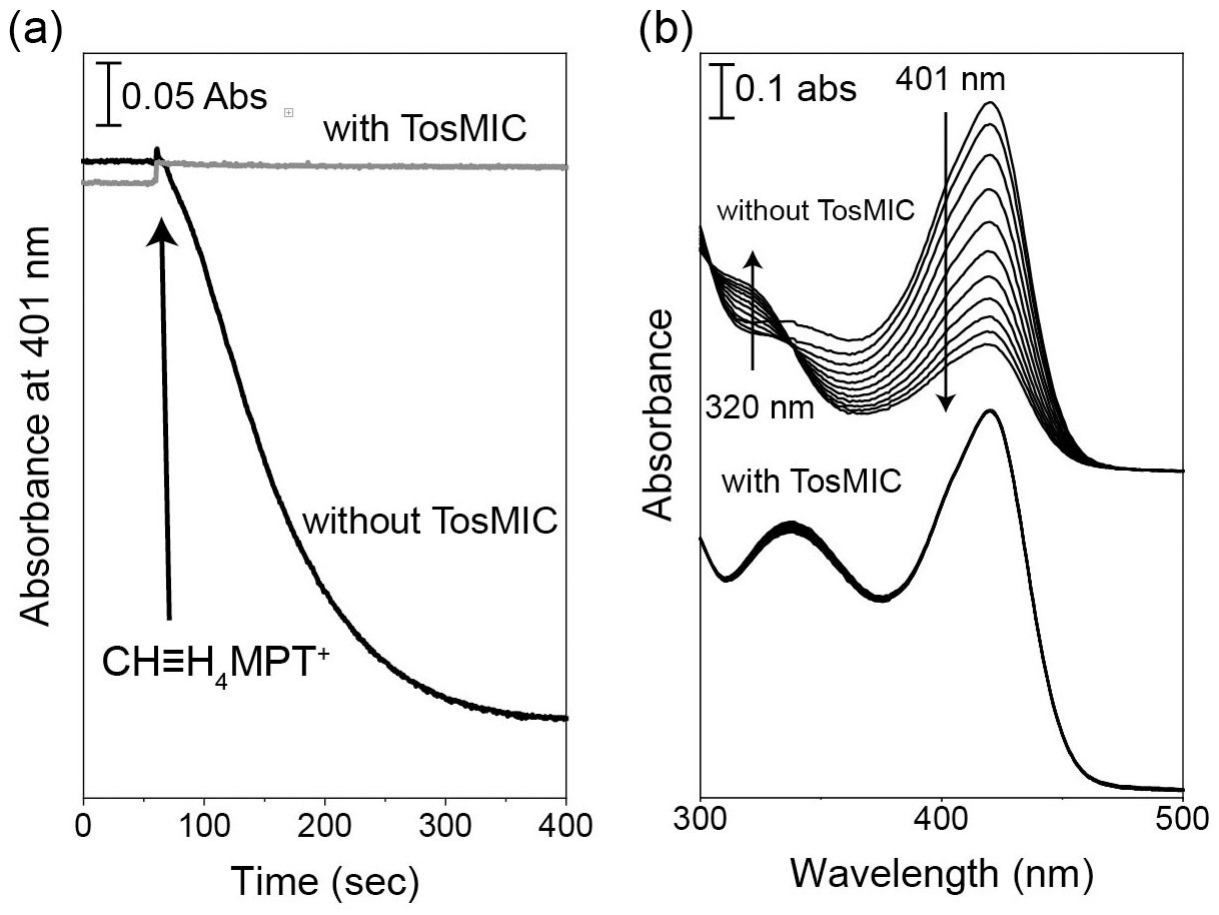
H_2_:F_420_‐reducing enzyme activity assay using the washed cell extract from the nickel‐limited culture (50 nM Ni^2+^) in the absence or presence of 10 µM TosMIC. (a) Kinetic record of absorbance at 401 nm: the reaction was started by the addition of methenyl‐H_4_MPT^+^ (CH≡H_4_MPT^+^), as shown by an arrow. The reduction of F_420_ was recorded by measuring the absorbance at 401 nm in accordance with standard assay conditions [12, 24]. (b) Time‐dependent changes of the UV‐vis spectra of the reaction shown in panel a. The directions of change for the peak at 320 nm and the shoulder at 401 nm with a reduction in F_420_ are shown by arrows.

In the previous reports, activity assays of cell extracts indicated that H_2_‐dependent F_420_‐reducing activity is mainly catalyzed by Frh under nickel‐sufficient conditions, whereas under nickel‐limited conditions, the Hmd+Mtd coupled reaction takes over the function of Frh [11, 12]. To confirm the contribution of Hmd to H_2_‐dependent F_420_‐reducing activity under nickel‐sufficient and nickel‐limited conditions, we investigated the effect of TosMIC, an Hmd inhibitor, on the reduction of F_420_ by cell extracts in an H_2_ atmosphere. In the cell extract from the methanogens cultivated under nickel‐sufficient conditions (5 µM Ni^2+^), no significant inhibition of the H_2_:F_420_‐reducing activity was observed upon the addition of TosMIC, which indicates that the H_2_:F_420_‐reducing activity in these cells was mainly catalyzed by Frh (Table 1). In contrast, in the methanogens cultivated under nickel‐limited conditions (50 nM Ni^2+^), the H_2_:F_420_‐reducing activity of cell extracts was abolished by the addition of TosMIC, which indicates that the H_2_:F_420_‐reducing activity in these cells was exclusively catalyzed by the Hmd+Mtd coupled reaction (Table 1). To further confirm this, we prepared the washed cell extract, in which most of the small compounds, including methenyl‐ and methylene‐H_4_MPT, were removed. The washed cell extract from nickel‐limited *M. marburgensis* cells did not show the H_2_:F_420_‐reducing activity; the addition of methenyl‐H_4_MPT^+^ was required to elicit this activity (Table 1 and Figure 2). In addition, the H_2_:F_420_ reduction activity within the washed extracts of nickel‐limited cells was inhibited by the addition of TosMIC. These experiments confirmed that the cell extract, which was derived from nickel‐limited cultures, catalyses H_2_‐dependent F_420_‐reducing activity only by the Hmd+Mtd coupled system.

**TABLE 1:** Activity assay of some enzymes related to F_420_ reduction with H_2_.

		Hmd^a^	Mtd^b^	H_2_:F_420_‐reducing activity	
				Without TosMIC	With TosMIC^b^
		Specific activity, U/mg			
5 µM Ni^2+^	Cell extract	1.6 ± 0.1	1.0 ± 0.1	3.2 ± 0.2	3.1 ± 0.6
50 nM Ni^2+^	Cell extract	8.9 ± 0.3	1.6 ± 0.1	0.16 ± 0.08^c^	ND^c^^,^ d
50 nM Ni^2+^	Washed cell extract	6.5 ± 0.4	1.8 ± 0.2	0.32 ± 0.03^c^	ND^c^^,^ d

a Reduction of methenyl‐H_4_MPT^+^ under H_2_ at pH 7.

b In the presence of TosMIC (1 µM for standard cell extracts and 10 µM for washed cell extracts) to inhibit Hmd.

c In the presence of 20 µM methenyl‐H_4_MPT^+^.

d Not detected (<0.005 U/mg). The average and standard error of three technical replicates are shown.

### 2.2 Difference in In Vitro Methanogenesis Activity Depending on Nickel Concentrations

**FIGURE 3:**
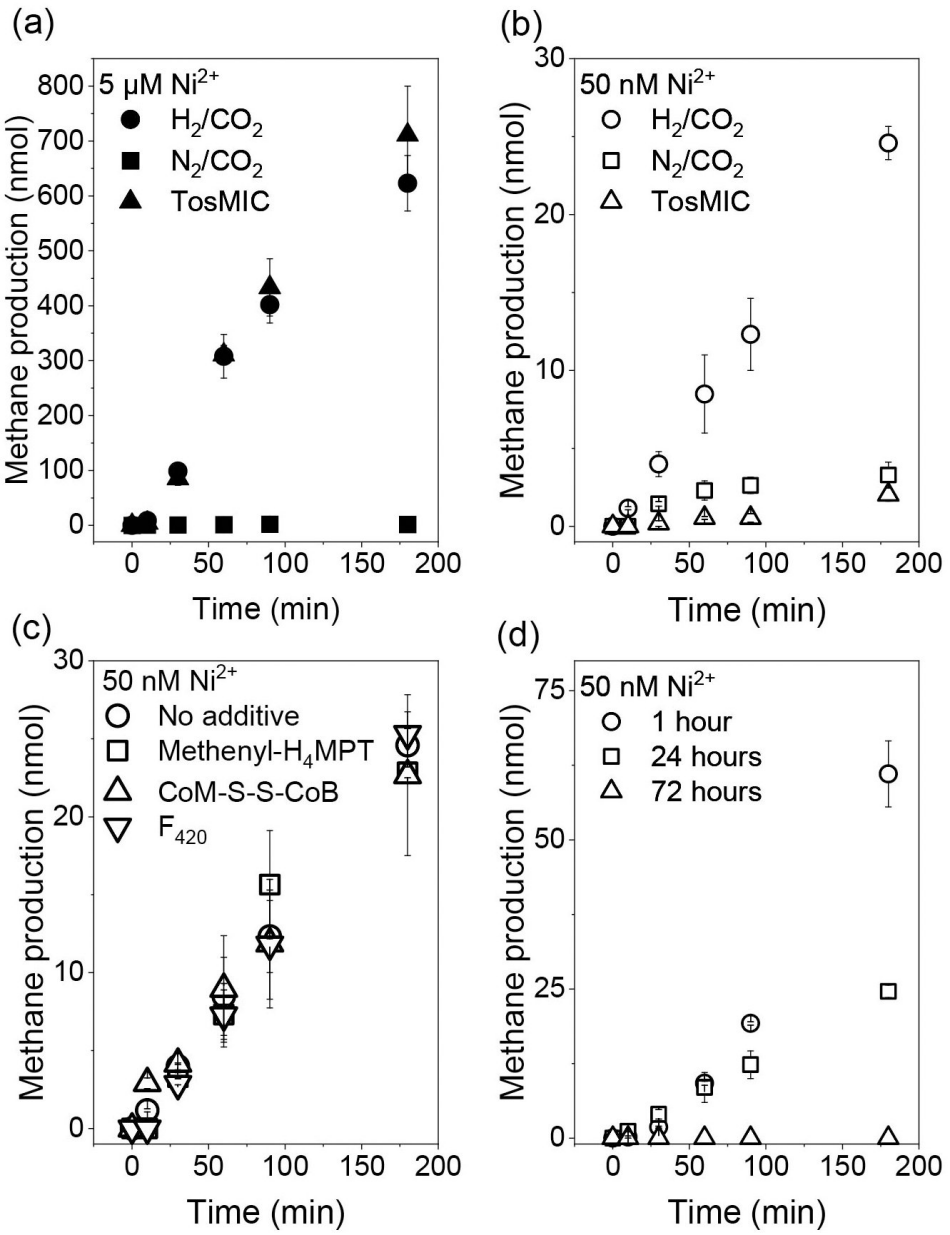
In vitro methanogenesis using the cell extract from nickel‐sufficient (5 µM Ni^2+^) and nickel‐limited (50 nM Ni^2+^) conditions. (a, b) Effect of 10 µM TosMIC. The data for a negative control without TosMIC under 80% H_2_/20%CO_2_ and 80% N_2_/20%CO_2_ are also shown. (c) Effect of addition of methanogenic coenzymes on in vitro methanogenesis using cell extract from nickel‐limited culture cells (50 nM Ni^2+^). (d) Time‐dependent inactivation of in vitro methanogenesis using cell extract from nickel‐limited culture cells on ice (50 nM Ni^2+^). Average and standard errors of two to four technical replicates from one biological replicate are shown.

To further confirm the role of Hmd in methanogenesis under nickel‐limited conditions, we established an in vitro assay to measure methanogenesis using cell extracts from *M. marburgensis* cells. Cell extracts from both nickel‐sufficient and nickel‐limited cells exhibited in vitro methanogenesis activity. However, the in vitro methanogenesis activity of the nickel‐limited culture cells was substantially (30‐fold) lower than that of nickel‐sufficient culture cells (Figure 3a, b). To test the effect of the key intermediates for the methanogenic reactions, we added methenyl‐H_4_MPT^+^, F_420_, and heterodisulfide (CoM‐S–S‐CoB) into the assay with the cell extract from nickel‐limited cells. The addition of these substances did not enhance the methanogenic activity (Figure 3c), which excluded the possibility that these intermediates were the limiting factor of the in vitro methanogenesis activity of the cell extract from the nickel‐limited cells. Notably, the methanogenic activity of the nickel‐limited sample decreased over time when incubated on ice after the cell extract was prepared (Figure 3d). In contrast, the in vitro methanogenesis assay of the cell extract obtained from the nickel‐sufficient condition (5 µM Ni^2+^) showed only a slight reduction in activity, even after 7 days of incubation (Figure S2). This suggests that the instability of the methanogenic enzymes in the cell extract from the nickel‐limited culture was one of the reasons for the observed low activity in the samples from the nickel‐limited condition. Under nickel‐limited conditions, the functions of the nickel‐containing hydrogenases Frh and Mvh were exchanged with the non‐nickel Hmd+Mtd‐coupled enzyme system and Elp [11]. Therefore, instability of the methanogenic reaction in the cell extract obtained under nickel‐limited conditions might be due to the possible instability of Hmd+Mtd and/or Elp, although in vivo instability of these enzymes has not been reported.

### 2.3 Inhibition of In Vitro Methanogenesis Activity With Cell Extracts by TosMIC

To evaluate the contribution of Hmd in methanogenesis, we determined the effect of TosMIC on the methanogenic activity of the *M. marburgensis* cell extract using the aforementioned in vitro methanogenesis assay in Section 2.2. TosMIC did not show a significant inhibitory effect on the methanogenic activity of the cell extract from the nickel‐sufficient condition (Figure 3a). In contrast, the addition of 10 µM TosMIC strongly inhibited the methanogenic activity of the cell extract from the nickel‐limited culture (Figure 3b). These data indicate that the Hmd+Mtd coupled enzyme system is crucial for methanogenesis in the cell extract from nickel‐limited culture cells, and TosMIC apparently does not inhibit methanogenic enzymes except for Hmd.

### 2.4 Inhibition of In Vivo Methanogenesis With Cell Suspensions by Isocyanides

**FIGURE 4:**
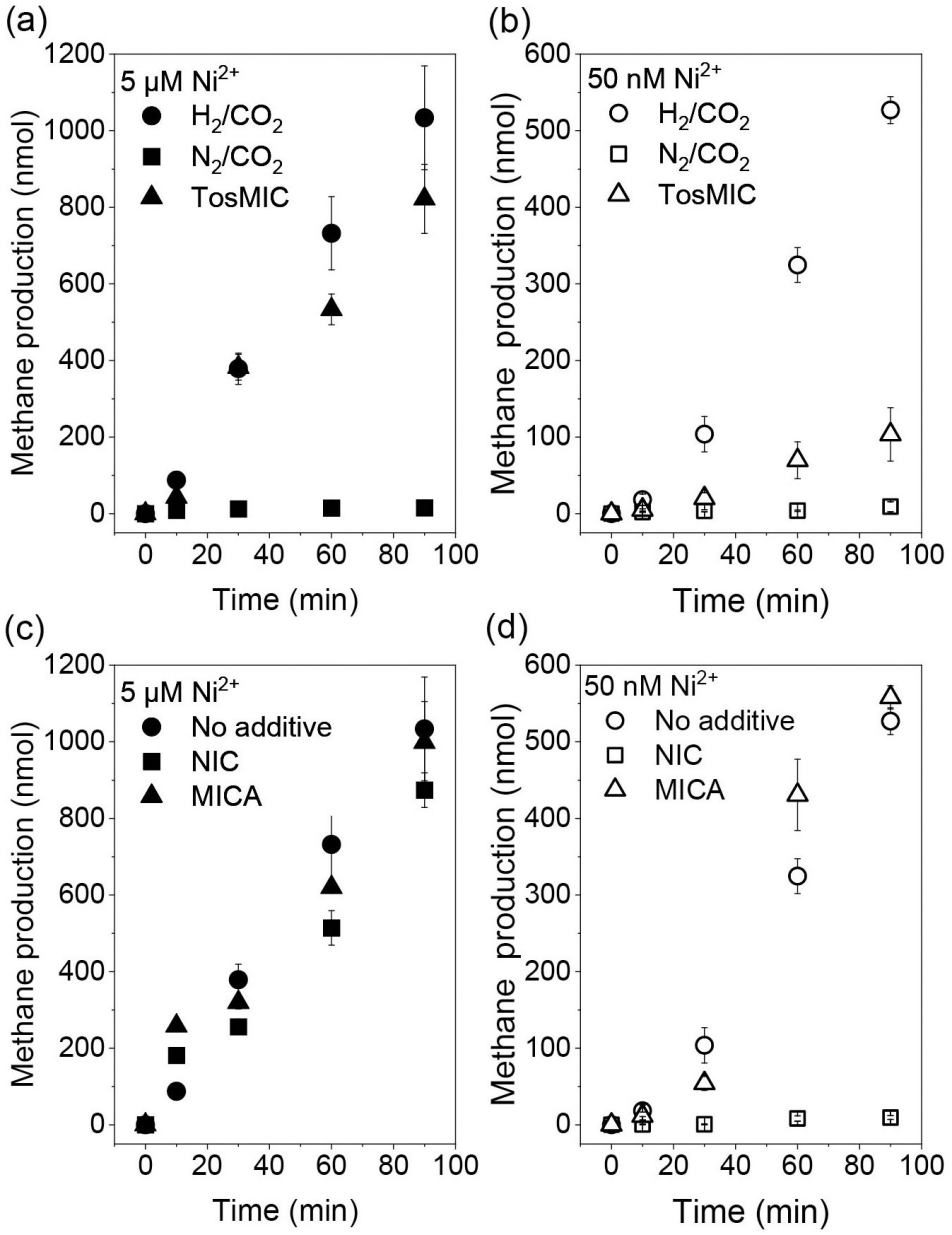
Inhibitory effect of isocyanides on in vivo methanogenesis using the cell suspension of *M. marburgensis* under 80% H_2_/20% CO_2_. (a, b) Effect of TosMIC on in vivo methanogenesis using the cell suspension from nickel‐sufficient (5 µM Ni^2+^) and nickel‐limited (50 nM Ni^2+^) conditions. Data for the negative control without TosMIC under 80% H_2_/20%CO_2_ and 80% N_2_/20%CO_2_ are also shown. (c, d) Effect of NIC and MICA. Data for a negative control without isocyanide (no additives) are also shown. The average and standard error of three technical replicates are shown.

To test its potential as a chemical tool for identifying in situ Hmd‐dependent methanogenesis activity in natural environmental samples, we determined whether the Hmd‐dependent pathway could be assessed in living cells using isocyanide inhibition. To this end, we conducted in vivo methanogenesis experiments using the cell suspension of *M. marburgensis*. The in vivo methanogenesis activity in the cell suspension obtained from culture conditions with sufficient nickel was twice as high as that obtained from nickel‐limited culture conditions. Such a difference in methanogenic activity is reasonable considering the ~two‐fold higher growth rate of the nickel‐sufficient culture [11]. In vivo methanogenesis with the cell suspension of nickel‐sufficient culture cells was slightly inhibited by TosMIC (Figure 4a). As in this condition, a certain amount of Hmd is produced [11, 12], it is likely that the slight decrease in methane production activity was attributable to the activity of Hmd in the cells from the nickel‐sufficient culture. In contrast, TosMIC (100 µM) substantially inhibited in vivo methanogenesis in the cell suspension of the nickel‐limited culture cells (Figure 4b). The observed partial inhibition of the nickel‐limited cells by TosMIC may be caused by the low permeability of this compound into the cells. To further test the inhibitory effect of isocyanides, we performed in vivo methanogenesis in the presence of hydrophobic NIC (100 µM) and hydrophilic methyl isocyanoacetate (MICA) (100 µM) (Figure 4c, d) (for the chemical structure of the isocyanides, see Scheme 1). MICA did not inhibit in vivo methanogenesis with a cell suspension of either the nickel‐sufficient or nickel‐limited culture cells. In contrast, NIC only exhibited strong inhibition when nickel‐limited cells were used. This result suggests that the permeability of isocyanides is crucial for these inhibitors to inhibit methanogenesis. These in vivo methanogenesis experiments using cell suspensions corroborate the results of experiments using cell extracts.

## 3 Conclusion

The detection of TosMIC‐sensitive and methenyl‐H_4_MPT^+^ dependent H_2_:F_420_‐reducing activity of the cell extract from the nickel‐limited culture and the inhibition of in vitro and in vivo methanogenesis by isocyanides support the previous proposal that the Hmd+Mtd coupled reaction is essential in the hydrogenotrophic methanogenesis in *M. marburgensis* under nickel‐limited conditions [11]. The strong inhibition of methanogenesis from the cell suspension indicates that isocyanides with a hydrophobic character (e.g., NIC) can be used to specifically inhibit methanogens utilizing Hmd as the major hydrogenase under nickel‐limited conditions. Unlike general methanogenesis inhibitors such as bromoethanesulfonate (BES), which suppress methanogenesis by inhibiting methyl‐coenzyme M reductase regardless of the metabolic pathway, isocyanides specifically target the Hmd‐dependent methanogenesis pathway. It should be noted that isocyanides may not inhibit other anaerobic microorganisms because active Hmd was only found in methanogenic archaea [25]. Therefore, isocyanides can serve as a selective diagnostic tool for identifying and evaluating the contribution of Hmd‐dependent methanogenesis in natural samples. These Hmd inhibitors will play a significant role in future research on methanogenesis metabolism in natural environments.

## 4 Experimental Section

### 4.1 Materials

*M. marburgensis* strain DSM2133 was purchased from the Leibniz Institute DSMZ‐German Collection of Microorganisms and Cell Cultures. Most chemicals were obtained from Sigma–Aldrich (Taufkirchen). We isolated H_4_MPT, methenyl‐H_4_MPT^+^ and F_420_ from *M. marburgensis* cells [26]. We prepared methylene‐H_4_MPT from H_4_MPT by reaction with formaldehyde [27]. We synthesized CoM‐S–S‐CoB according to the previous reports [18, 21, 28].

### 4.2 Preparation of Cell Extract and Enzyme Assay

*M. marburgensis* was cultivated by continuous cultivation under nickel‐sufficient (5 µM Ni^2+^) and nickel‐limited condition (50 nM Ni^2+^) and harvested as described in the previous report [11]. Cell extract was anaerobically prepared in an anaerobic chamber [11]. The cells were suspended in 50 mM Tris/HCl pH 7.6 containing 2 mM dithiothreitol, and the suspension was ultrasonicated on ice/water for 2 min using a SONOPULS GM200 (Bandelin) with a 72D tip with 30% cycles 12 times, with 2‐min breaks between sonication cycles. The sample was anaerobically centrifuged with a Beckman JA‐25.50 at 13,000×*g* at 4 °C for 30 min. We measured the activity of F_420_ reduction with H_2_ (H_2_:F_420_‐reducing activity) according to a previous report [12]. We determined Hmd activity by recording methenyl‐H_4_MPT^+^ reduction under H_2_ (+0.4 bar) in 120 mM potassium phosphate buffer containing 1 mM EDTA according to the previous report, with a different pH value (7.0 instead of 7.5 in the previous report) [5, 27].

We determined the Hmd+Mtd‐coupled enzyme activity in the washed cell extract by recording the reduction of F_420_ under H_2_ (+0.4 bar) in the presence of methenyl‐H_4_MPT^+^. To prepare the washed cell extract, we removed small components in the cell extract through concentration and dilution cycles using a 10 kDa ultrafilter. We preincubated 120 mM potassium phosphate buffer pH 7.0 with 1 mM EDTA (0.7 mL) at 40 °C for 5 min in a 1 cm light‐path quartz cuvette (1 mL), and then we added 10 µL of 1.4 mM F_420_ (final concentration of 20 µM) and 10 µL of the washed cell extract to the cuvette. Adding 10 µL of 1.4 mM methenyl‐H_4_MPT^+^ (final concentration of 20 µM) started the reaction. One unit was defined as the reduction of 1 µmol of F_420_ per minute (the extinction coefficient of F_420_ at 401 nm: 25.9 mM^−1^cm^−1^). The 1 µM and 10 µM concentrations of TosMIC were used for the assays with cell extracts and washed cell extract, respectively.

### 4.3 In Vitro Methanogenesis Experiments

In vitro methanogenesis experiments with the cell extract were performed under almost identical conditions to those in the previous report [29], except for the addition of ATP (for this reason, see below). For the preparation of the cell extract, the cell pellet (3 wet g) was suspended in 10 mL of 100 mM Pipes/NaOH pH 6.2 containing 15 mM MgCl_2_ and 10 mM 2‐mercaptoethanol according to a previous publication [29]. We diluted the supernatant with 1.5 mL of the same buffer solution to 2 mg/mL protein. The diluted cell extract (200 µL) was injected into an amber vial with a rubber stopper filled with N_2_ and incubated at 65 °C for 5 min. Exchanging the gas phase from 100% N_2_ to 80% H_2_/20% CO_2_ started the reaction. The *M. marburgensis* cell extract exhibited in vitro methanogenesis even in the absence of pre‐incubation with ATP, albeit at a reduced rate compared to the previous studies using the *M. thermautotrophicus* strain Δ*H* [29]. In our in vitro methanogenesis experiments in the presence of 5 mM ATP, the methanogenic reaction started with no activity for around 60 min, after which it gradually increased. The final rate of the methanogenic reaction was similar to that in the absence of ATP. ATP appears to have an inhibitory effect on the methanogenic reactions of the *M. marburgensis* cell extract. Wolin et al. also observed that although methanogenesis by *M. thermautotrophicus* Δ*H* requires ATP, higher concentrations of it have an inhibitory effect [30]. To measure the methane content, the gas phase (0.1 mL) was injected into a gas chromatograph (Porapak Q, 80/100 mesh, 274 cm by 3.18 mm). The carrier gas was N_2_. H_2_ was used as the fuel gas for the flame ionization detector. Calibration of the instrument was conducted using standard gases (1%, 5%, and 10% CH_4_ in 100% N_2_).

For in vivo methanogenesis from the cell suspension, the fresh cell pellet was suspended in a culture medium, in which the optical density at 600 nm of the cell suspension was adjusted to 1.5, equivalent to 0.2 mg/mL protein in the cell suspension. The cell suspension (200 µL) was injected into an amber vial with a rubber stopper filled with N_2_ and incubated at 65 °C for 5 min. To start the reaction, the gas phase was exchanged with 80% H_2_/20% CO_2_ and incubated at 65 °C (water bath). The CH_4_ content was measured using gas chromatography as described above.

## Funding

This work was supported by Max‐Planck‐Gesellschaft, Deutsche Forschungsgemeinschaft, and Asahi Kasei Pharma Corporation.

## Conflicts of Interest

The authors declare no conflicts of interest.

## Supporting information

Supplementary Material

## Data Availability

The data that support the findings of this study are available in the Supporting Information of this article.
