# Supplementary material for: Isocyanides as a Chemical Tool for Identifying [Fe]‐Hydrogenase‐Dependent Methanogenesis
Source: Chembiochem. 2026 Sep 14;27(17):e70535. doi: 10.1002/cbic.70535 (PMC13572956; doi:10.1002/cbic.70535)
Supplement: Supplementary file 1 [file CBIC-27-e70535-s001.pdf]

Supporting Information for

Isocyanides as a Chemical Tool for Identifying [Fe]-Hydrogenase-Dependent Methanogenesis

Shunsuke Nomura and Seigo Shima*
E-mail: shima@mpi-marburg.mpg.de

This document includes:
Figures S1 and S2

The authors have cited no additional references within the Supporting Information.

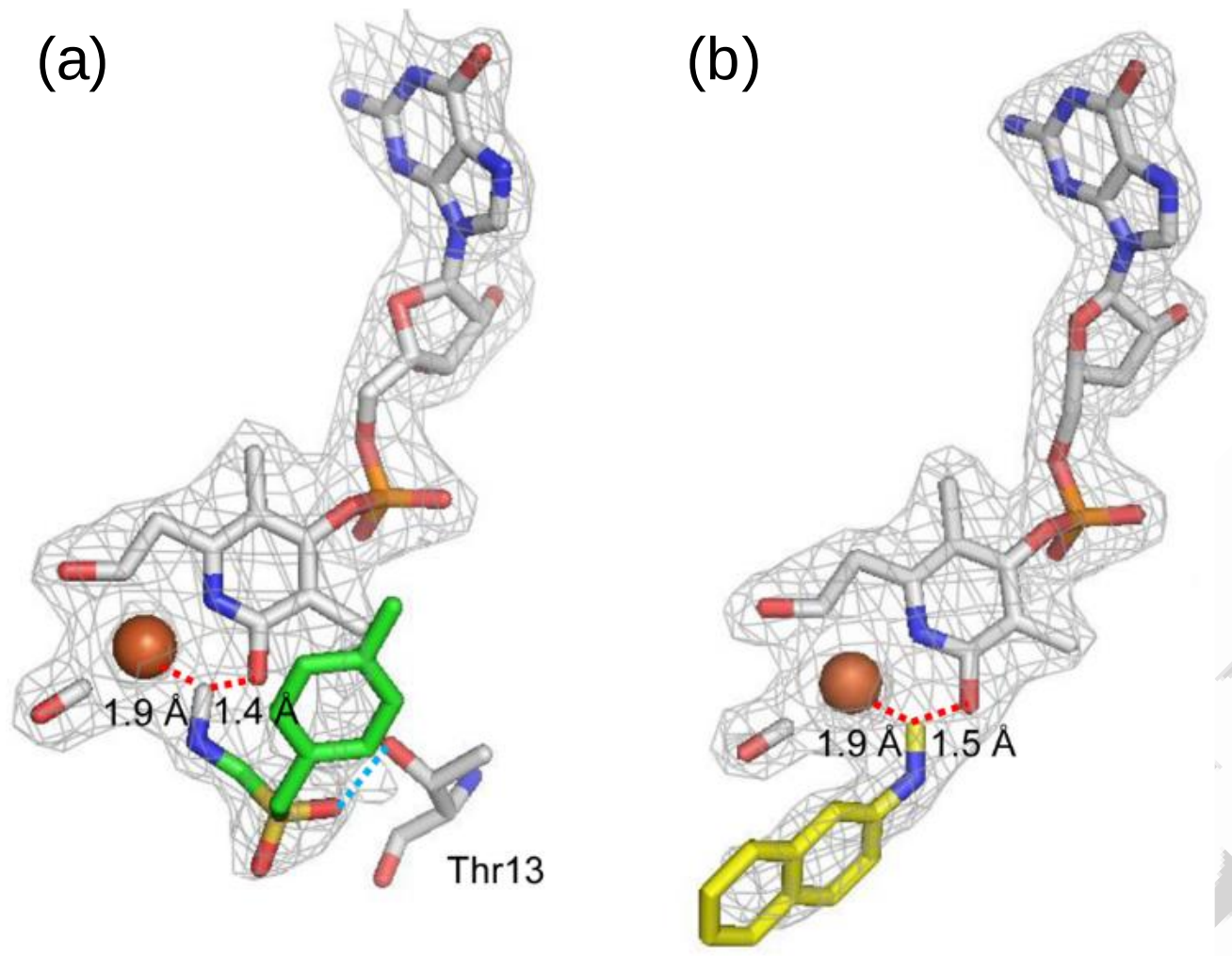

Figure S1. Binding of isocyanide inhibitors to the FeGP cofactor of [Fe]-hydrogenase. a) TosMIC and b) NIC with FeGP cofactors as stick models. The C atoms of the FeGP cofactor, TosMIC, and NIC are drawn in light gray, green, and yellow; O red, N blue, S yellow, Fe brown sphere. Covalent bonds between FeGP and the inhibitors are red dashed lines. A potential hydrogen bond between the sulfonyl O atom of TosMIC and the hydroxy group of Thr13 is a blue dashed line. The $2F_o–F_c$ electron density maps (in gray mesh) are contoured at 1.0σ. This figure is reproduced with permission from [8].

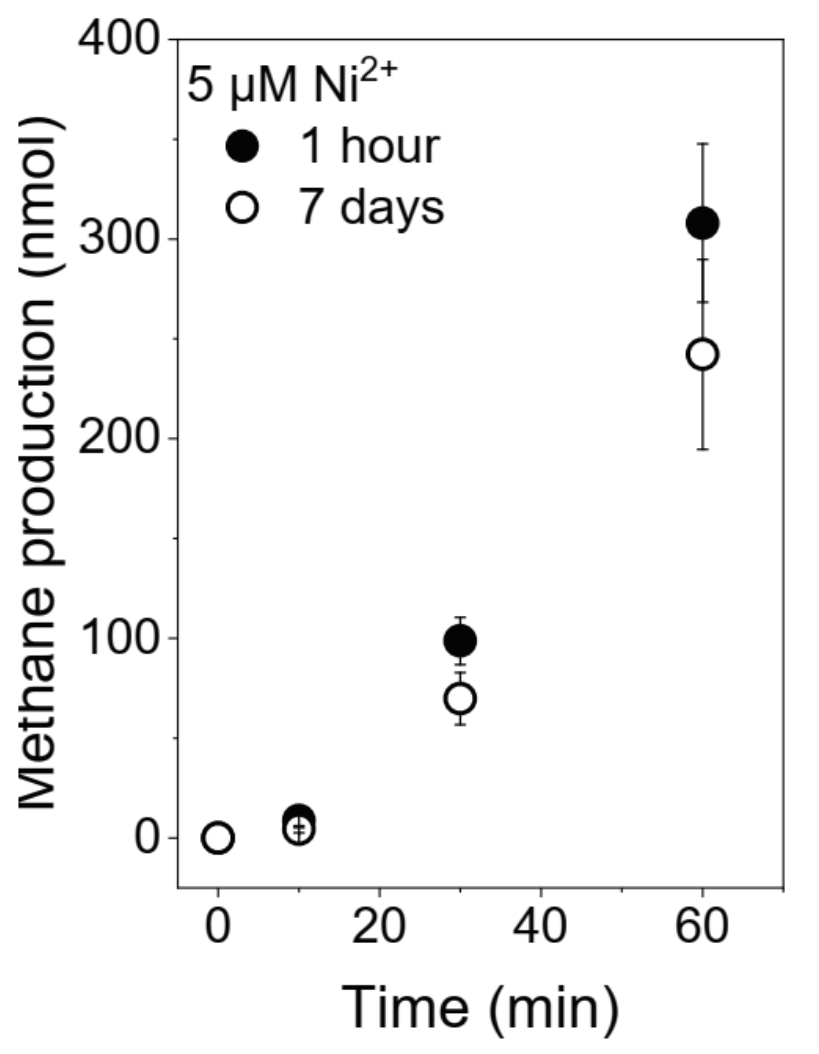

Figure S2. Time-dependent inactivation of in vitro methanogenesis using cell extract from nickel-sufficient culture cells (5 µM Ni^{2+}). Incubation time on ice after preparation of cell extract is indicated. The 1-h and 7-d data are a result of the average and standard error of four and two technical replicates from one biological replicate, respectively.
